# MiR-451 inhibits cell growth and invasion by targeting *MIF* and is associated with survival in nasopharyngeal carcinoma

**DOI:** 10.1186/1476-4598-12-123

**Published:** 2013-10-20

**Authors:** Na Liu, Ning Jiang, Rui Guo, Wei Jiang, Qing-Mei He, Ya-Fei Xu, Ying-Qin Li, Ling-Long Tang, Yan-Ping Mao, Ying Sun, Jun Ma

**Affiliations:** 1 Sun Yat-sen University Cancer Center; State Key Laboratory of Oncology in South China; Collaborative Innovation Center for Cancer Medicine, 651 Dongfeng Road East, Guangzhou People’s Republic of China

**Keywords:** miR-451, MIF, Cell growth, Invasion, Survival, Nasopharyngeal carcinoma

## Abstract

**Background:**

MiRNAs play important roles in diverse biological processes including tumorigenesis. However, little is known about the function and mechanism of miR-451 in nasopharyngeal carcinoma (NPC).

**Methods:**

Quantitative RT-PCR was used to quantify miR-451 expression in NPC cell lines and clinical tissues. Kaplan-Meier curves were used to estimate the association between miR-451 expression and survival. The MTT, colony formation, Transwell migration and invasion assays, and a xenograft model were performed. A miR-451 target was confirmed using luciferase reporter assays, quantitative RT-PCR, and Western blotting.

**Results:**

MiR-451 was significantly downregulated in NPC cell lines and clinical tissues (*P* < 0.01). Patients with low expression of miR-451 had poorer overall survival (HR, 1.98; 95% CI, 1.16-3.34; *P* = 0.01) and disease-free survival (HR, 1.68; 95% CI, 1.07-2.62; *P* = 0.02) than patients with high expression. MiR-451 was an independent prognostic factor in NPC in multivariate Cox regression analysis. Ectopic expression of miR-451 suppressed cell viability, colony formation, and cell migration and invasion in vitro, and inhibited xenograft tumor growth in vivo. *MIF* was verified as a direct target of miR-451, and MIF regulated NPC cell growth and invasion.

**Conclusions:**

The newly identified miR-451/*MIF* pathway provides insight into NPC initiation and progression, and may represent a novel therapeutic target.

## Background

Nasopharyngeal carcinoma (NPC) is a common head and neck cancer derived from epithelium cells located in the nasopharynx. The global statistics by world region revealed its extremely unbalanced endemic distribution, with the highest incidence in Southern China [1]. Although advances made in clinical treatment, the prognosis of NPC patients, especially with advanced disease, is still very poor due to the recurrence and distant metastasis [2]. Genetic susceptibility, Epstein-Barr virus infection, and environmental factors have been reported to be the major etiologic factors of NPC [3]. Up to date, the accurate molecular mechanism underlying the pathogenesis and progression of NPC is still not fully understood. Therefore, a better understanding of the molecular mechanism involved in NPC progression is essential for the development of novel therapeutic strategies for NPC patients.

MicroRNAs (miRNAs) are small non-coding RNA molecules about 19–25 nucleotide, which exist in many organisms and regulate gene expression at the post-transcriptional level by base pairing with the 3′-untranslated region (3′-UTR) of their target genes [4-6]. Generally, a miRNA can regulate multiple target genes and one gene may be regulated by multiple miRNAs, underscoring the formation of complex regulatory networks [4]. It has been reported that miRNAs can control a variety of biological processes including cellular differentiation, proliferation, and apoptosis [7-9]. Recent evidence also indicated that miRNAs may function as tumor suppressors or oncogenes, and play critical roles in carcinogenesis [10-12]. Inhibiting or reconstituting special miRNA may have therapeutic implication and makes them candidates for therapeutic targets [13-16]. Antagomirs are a type of antisense oligonucleotide that can effectively inhibit miRNAs function in vivo [13,14]. Dysregulated expression of miRNAs has been reported in most tumor types [17-20], including NPC [21-23]. Recent studies also reported that several dysregulated miRNAs involved in NPC development and progression by regulating cell growth, proliferation, apoptosis, invasion, and metastasis [24-27], indicating that miRNAs play important roles in NPC tumorigenesis. In our recent microarray study, we found that miR-451 was downregulated in NPC [23], however, no study has elucidated the functions and mechanisms of miR-451 in NPC development and progression.

In this study, we investigated the prognostic value and potential roles of miR-451 in NPC. We demonstrated that miR-451 was downregulated in NPC cell lines and tissue samples. We further explored its effects on cell growth, colony formation, invasive, and tumorigenesis. Moreover, macrophage migration inhibitory factor (MIF) was identified as a functional target of miR-451. The newly identified miR-451/MIF pathway elucidated the roles of miRNAs in the development of NPC and would provide a novel therapeutic strategy for NPC.

## Results

### MiR-451 is downregulated in NPC cell lines and clinical specimens

**Figure 1:**
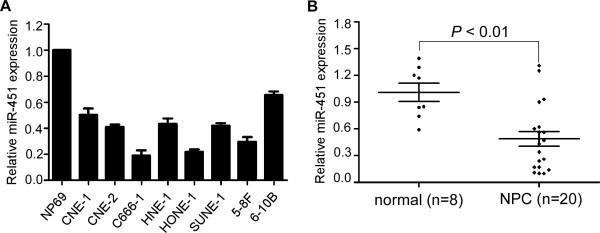
**MiR-451 is downregulated in NPC cell lines and clinical samples. (A)** Relative expression of miR-451 in NP69 and NPC cell lines. **(B)** Relative expression of miR-29c in NPC (*n* = 20) and normal nasopharyngeal epithelial tissues (*n* = 8). U6 used as the endogenous control. Data is presented as the mean ± SD, and *P* values were calculated with the Student’s *t*-test.

In this study, we firstly tested miR-451 expression in NPC cell lines and the normal nasopharyngeal epithelial cell line NP69, and found it was significantly decreased in NPC cell lines (Figure 1A). We also detected miR-451 expression in 20 freshly-frozen NPC and 8 normal nasopharyngeal epithelial tissue samples and found that the miR-451 expression was significantly downregulated in NPC tissues (Figure 1B, *P* < 0.01).

### Downregulation of miR-451 is associated with worse survival in NPC

**Figure 2:**
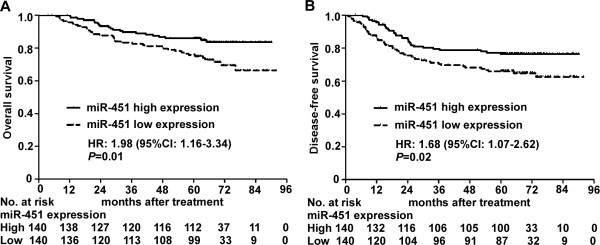
**Downregulation of miR-451 is associated with worse survival in NPC patients. (A)** Overall survival. **(B)** Disease-free survival. HR, hazard ratio, and CI, confidence interval; HR values were calculated with the unadjusted Cox regression analysis, and *P* values were calculated with the log-rank test.

To evaluate the clinical significance of miR-451, we detected miR-451 expression in a cohort of 280 paraffin-embedded NPC biopsy samples using quantitative RT-PCR. Patients with low expression of miR-451 were found to have significantly worse overall survival (HR, 1.98; 95% CI, 1.16-3.34; *P* = 0.01) and disease-free survival (HR, 1.68; 95% CI, 1.07-2.62; *P* = 0.02) than those with high expression (Figure 2). However, no significant correlations were found between miR-451 expression and any clinical characteristics (Table 1). In addition, multivariate Cox regression analysis showed that miR-451 expression and clinical stage were both independent prognostic indicators for overall survival (HR, 2.00; 95% CI, 1.18-3.41; *P* = 0.01; HR, 2.93; 95% CI, 1.44-5.97; *P* < 0.01) and disease-free survival (HR, 1.81; 95% CI, 1.16-2.83; *P* = 0.01; HR, 2.41; 95% CI, 1.37-4.23; *P* < 0.01).

**Table 1:** Associations between miR-451 expression and clinical characteristics in patients with nasopharyngeal carcinoma

**Characteristics**	**No. of patients**	**Expression of miR-451**		***P*value**
		**Low, n (%)**	**High, n (%)**	
**Age**				
≤ 45 yr	136	66 (47)	70 (50)	0.63
> 45 yr	144	74 (53)	70 (50)	
**Sex**				
Male	206	100 (71)	106 (76)	0.42
Female	74	40 (29)	34 (24)	
**WHO Type**				
I + II	11	4 (3)	7 (5)	0.36
III	269	136 (97)	133 (95)	
**VCA-IgA**				
< 1:80	43	21 (15)	22 (16)	0.87
≥ 1:80	237	119 (85)	118 (84)	
**EA-IgA**				
< 1:10	71	37 (26)	34 (24)	0.68
≥ 1:10	209	103 (74)	106 (76)	
**T Stage**				
T1-T2	142	70 (50)	72 (51)	0.81
T3-T4	138	70 (50)	68 (49)	
**N Stage**				
N0	42	89 (64)	85 (61)	0.62
N1-N3	238	51 (36)	55 (39)	
**TNM Stage**				
I-II	91	49 (35)	42 (30)	0.37
III-IV	189	91 (65)	98 (70)	

VCA-IgA: viral capsid antigen immunoglobulin A; EA-IgA: early antigen immunoglobulin A. All patients were restaged according to the 7th edition of the AJCC Cancer Staging Manual.

### MiR-451 suppresses NPC cell viability and colony formation in vitro

**Figure 3:**
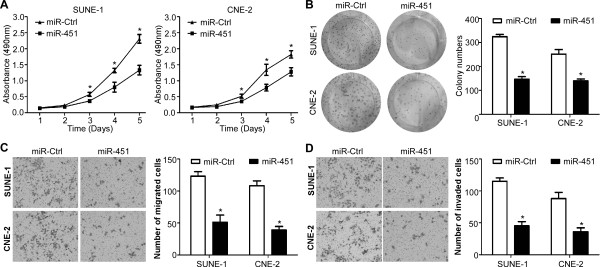
**miR-451 suppresses NPC cell viability, colony formation, migration and invasion. (A)** MTT assay was performed to test cell viability of SUNE-1 and CNE-2 cells. **(B-D)** Representative results of colony formation **(B)**, migration **(C)**, and invasive ability **(D)** of SUNE-1 and CNE-2 cells transfected with miR-29c mimics or miR control. Data is presented as the mean ± SD. *, *P* < 0.01 compared with control using the Student’s *t*-test.

To explore whether ectopic expression of miR-451 affects cell viability and proliferation ability, MTT assay and colony formation assay were performed after transient transfection with miR-451 mimics or miR controls in SUNE-1 and CNE-2 cells. Cells transfected with miR-451 mimics showed a significant inhibition of growth compared with those transfected with miR controls (Figure 3A, *P* < 0.01). Moreover, cells transfection with miR-451 mimics displayed much fewer and smaller colonies compared with controls (Figure 3B, *P* < 0.01).

### MiR-451 suppresses NPC cell migration and invasion in vitro

To determine whether ectopic expression of miR-451 has a biological affect on the cell migration and invasive ability, SUNE-1 and CNE-2 cells were transiently transfected with miR-451 mimics or miR controls. Transwell migration assay demonstrated that the migration ability of cells transfected with miR-451 mimics was much lower than those transfected with miR controls (Figure 3C, *P* < 0.01). Transwell invasion assay also showed that transfection of miR-451 mimics significantly reduced the invasive ability (Figure 3D, *P* < 0.01).

### MiR-451 suppresses NPC xenograft tumor growth in vivo

**Figure 4:**
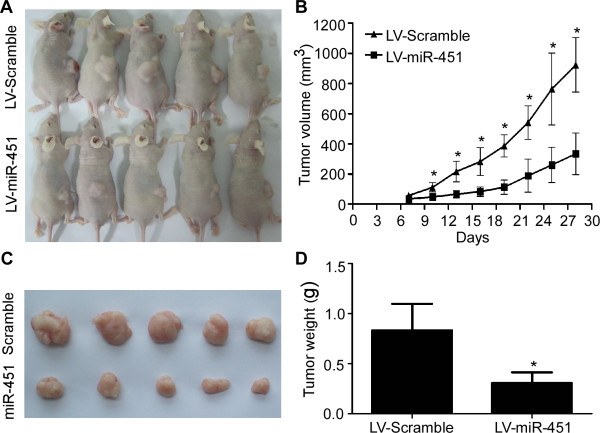
**miR-451 suppresses NPC xenograft tumor growth in vivo. (A)** SUNE-1 cells stably overexpressing miR-451 or scrambled miRNA was subcutaneously injected into nude mice. Four weeks later, SUNE-1 cells stably overexpressing miR-451 had smaller tumors than controls. **(B)** The growth curves of tumor volumes. **(C)** Representative picture of tumors formed. **(D)** Tumor weight. Data is presented as the mean ± SD. *, *P* < 0.01 compared with control using the Student’s *t*-test.

To further explore whether ectopic expression of miR-451 affects tumor growth in vivo, we conducted xenografts tumor model assay by subcutaneously injecting SUNE-1 cells stably overexpressing miR-451 or scrambled miRNA in the dorsal flank of nude mice. We found that the tumors in the group injected with SUNE-1 cells stably overexpressing miR-451 grew at a slower rate and had smaller volumes than the scrambled control (Figure 4A-B, *P* < 0.01). The average tumor weight was also significantly lower in the miR-451 overexpressing group (0.31 ± 0.11 g vs. 0.83 ± 0.26 g; Figure 4C-D, *P* < 0.01).

### MIF is a direct target of miR-451 and involved in NPC cell growth and invasion

**Figure 5:**
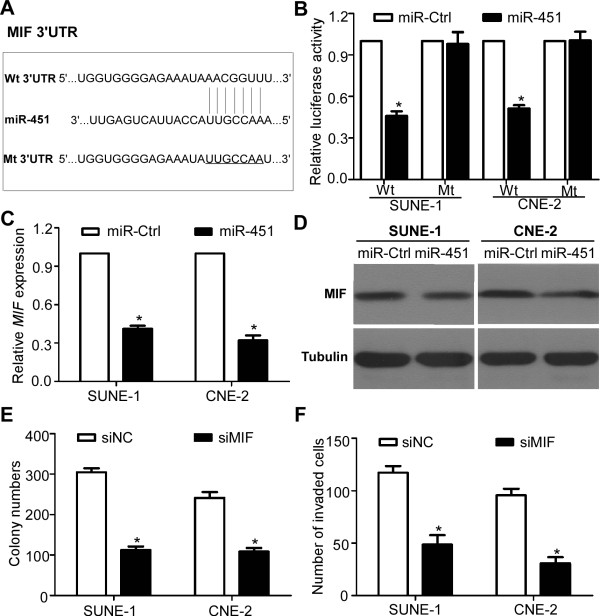
***TIAM1 *is a direct target of miR-29c and involved in NPC cell growth and invasion. (A)** Wild-type or mutant miR-451 target sequences of *MIF* 3′ UTR. **(B)** Relative luciferase activity of SUNE-1 and CNE-2 cells after co-transfection with wild type (Wt) or mutant (Mt) *MIF* 3′ UTR reporter genes and miR-29c mimics or control. **(C, D)** Quantification of *MIF* mRNA **(C)** and protein **(D)** expression after transfection with miR-451 mimics or control. **(E, F)** Colony formation **(E)** and invasive ability **(F)** after transfection with siMIF or control. Data is presented as the mean ± SD. *, *P* < 0.01 compared with control using the Student’s *t*-test.

To address the molecular mechanism by which miR-451 suppressed NPC cell growth and invasion, we identified *MIF* as a potential target of miR-451 using two publicly available databases (TargetScan and DIANA). We constructed luciferase reporter vectors that contained wild-type or mutant miR-451 target sequences of the *MIF* 3′ UTR (Figure 5A), and performed luciferase reporter assay to determine whether *MIF* was a direct target of miR-451. We found that ectopic expression of miR-451 inhibited the luciferase activity of the wild-type 3′ UTR reporter gene but not the mutant reporter gene (Figure 5B, *P* < 0.01), indicating that miR-451 can bind to the 3′ UTR of *MIF*. Furthermore, we found that ectopic expression of miR-451 could suppress the mRNA and protein expression of *MIF* (Figure 5C-D, *P* < 0.01). Finally, to determine whether MIF could regulate cell growth and invasion, we transiently transfected SUNE-1 and CNE-2 cells with siMIF or siRNA controls, and found that siMIF reduced the colonies formation and suppressed invasive ability (Figure 5E-F, *P* < 0.01).

## Discussion

In this study, we found that miR-451 was downregulated in NPC cell lines and clinical samples, and downregulation of miR-451 was associated with worse survival in patients with NPC. Ectopic expression of miR-451 suppressed NPC cell growth and invasion in vitro and inhibited tumor growth in vivo. Furthermore, MIF was verified as a direct target of miR-451, and involved in NPC cell growth and invasion. Our results suggest that the downregulation of miR-451 has important roles in the development and progression of NPC.

Recently, miRNAs have been shown to be important in maintenance of normal cellular function, and the dysregulation of miRNAs expression can result in cancer initiation and tumor progression [7,10,11]. Several studies reported that miRNAs were dysregulated in NPC [21-23], and the dysregulated miRNAs could regulate NPC cell growth, proliferation, and metastasis [24-27]. In our recent microarray study, we found that miR-451 was significantly reduced in NPC. However, little is known about the function and mechanism of miR-451 involving in NPC development and progression. Our results further demonstrated that miR-451 was downregulated in NPC cell lines and freshly-frozen tissue samples. Strikingly, we found that the downregulation of miR-451 was significantly associated with worse survival in patients with NPC. Multivariate Cox regression analysis further demonstrated that low expression of miR-451 was an independent prognostic indicator for NPC patients. These results indicated that miR-451 could be a useful prognostic biomarker to stratify NPC patients into different risk groups and further guide the personalized therapy for NPC patients.

MiR-451 was reported to be frequently downregulated in several types of tumors [28], such as gastric cancer [29], lung cancer [30], glioma [31,32], and breast cancer [32]. Moreover, the dysregulation of miR-451 expression was involved in the carcinogenesis and progression by affecting the tumor cellular function, including cell proliferation and growth, cell-cycle distribution, migration, and invasion [29-31]. In this study, to better understanding the function of miR-451 in NPC, we firstly detected the effect of miR-451 on cell viability and colony formation using MTT assay and colony formation assay. The results showed that ectopic expression of miR-451 could significantly suppress the cell viability and colony formation ability. Furthermore, the Transwell migration and invasion assays were conducted, and the results demonstrated that ectopic expression of miR-451 could inhibit the cell migration and invasive ability. We also found that ectopic miR-451 inhibited the xenograft tumor growth in vivo. Taking together, these results suggested that the regulation of miR-451 on cell growth and invasion may contribute the development and progression of NPC.

Each miRNA has the potential to target hundreds of genes that harbor target sequence in their 3′ UTR complementary to the seed region of the miRNA [4]. Several targets of miR-451, such as calcium binding protein 39 (CAB39) [32], ras-related protein 14 (RAB14) [30], and 14-3-3§ [33], have been identified. In our present report, we verified *MIF* as a direct target of miR-451 using luciferase reporter gene assay, which was consistent with the finding in gastric cancer [29]. Furthermore, ectopic expression of miR-451 could significantly reduce the *MIF* expression at both the mRMA and protein levels. Several studies have reported that the expression of MIF was upregulated in malignant tumors [34], and correlated with its aggressiveness and metastatic potential by promoting cell growth and invasion [35,36]. Studies also demonstrated that increasing expression of MIF protein was associated with poor prognosis in NPC patients [37,38]. In this study, we found that siMIF could significantly inhibit the colonies formation and invasive ability of NPC cells. These findings suggested that miR-451 suppressed the cell growth and invasion by targeting MIF.

## Conclusions

In conclusion, this report found that downregulation of miR-451 was associated with worse survival in patients with NPC, and ectopic expression of miR-451 could suppress cell growth and invasion by directly targeting *MIF*. This newly identified miR-451/MIF pathway provides new insight into the molecular mechanisms which regulate NPC progression, and further provides novel therapeutic strategies for NPC patients.

## Materials and methods

### Clinical specimens and cell culture

A total of 280 paraffin-embedded NPC specimens were obtained from Sun Yat-sen University Cancer Center between January 2003 and February 2006. Twenty freshly-frozen NPC samples and eight normal nasopharyngeal epithelium samples were also collected from our center. No patients had received any anti-tumor treatments before biopsy. The clinical staging was reclassified according to the 7th edition of the AJCC Cancer Staging Manual. All patients were treated with conventional two-dimensional radiotherapy and 151 (79.9%) of 189 patients with advanced disease (T_3_-T_4_ or N_2_-N_3_) also received platinum-based induction or concomitant chemotherapy. The clinical characteristics of NPC patients were listed in Table 1. The median follow-up time was 63.9 months (range: 3.7-91.87). Written informed consent was obtained from each patient for the use of their biopsy samples, and the research protocols were approved by the Academic Committee of Sun Yat-sen University Cancer Center of our hospital.

The human immortalized nasopharyngeal epithelial cell line NP69 was maintained in Keratinocyte/serum-free medium (Invitrogen) supplemented with bovine pituitary extract (BD Biosciences), human NPC cell lines (CNE-1, CNE-2, C666-1, HNE-1, HONE-1, SUNE-1, 5-8 F, and 6-10B) were cultured in RPMI-1640 (Invitrogen) supplemented with 10% FBS (Gibco), and 293FT cells were grown in DMEM (Invitrogen) supplemented with 10% FBS.

### RNA extraction, reverse transcription, and real-time RT-PCR

Total RNA was extracted from paraffin-embedded samples with acid phenol-Chloroform method [39], and from freshly-frozen samples with TRIzol reagent (Invitrogen). Total RNA was reverse-transcribed with M-MLV reverse transcriptase (Promega) and Bulge-Loop™ miRNA specific RT-primers (RiboBio) for miR-451 or random primers (Promega) for *MIF*. Real time PCR reactions were conducted using Platinum SYBR Green qPCR SuperMix-UDG reagents (Invitrogen) on the PRISM 7900HT system (Applied Biosystems). All reactions were done in triplicate and reactions without reverse transcriptase were used as negative controls. The U6 or GAPDH were used as the endogenous controls for miR-451 or *MIF* and the 2^-ΔΔCT^ equation was used to calculate the relative expression levels [40].

### Oligonucleotide transfection and generation of stably transfected cell lines

Cells were seeded into 6-well plates, transfected with miR-451 mimics or miR controls (50 nM, GenePharma) using Lipofactamine™ RNAiMAX (Invitrogen) and transfected with siMIF (100 nM, Invitrogen) or siRNA controls using Lipofactamine 2000 reagent (Invitrogen), and then harvested for assays 48 h later. The lentiviral plasmid pEZX-MR03 (GeneCopoeia) expressing miR-451 (Cat, HmiR0274-MR03) or scrambled miRNA (Cat, CmiR0001-MR03) and Lenti-Pac HIV Expression Packaging mix (GeneCopoeia) were cotransfected into 293FT cells using EndoFectin Lenti transfection reagent (GeneCopoeia). After transfection for 48 h, lentiviral particles were harvested and then transduced into the SUNE-1 cells, and the stably transfected cells were selected using puromycin and validated by real time RT-PCR.

### MTT assay and colony formation assay

SUNE-1 and CNE-2 cells were seeded at 1500 cells per well in 96-well plates after transfection. MTT assay was performed to test cell viability at 1, 2, 3, 4, and 5 days, and the absorbance was measured at 490 nm with a spectrophotometric plate reader. For colony formation assay, SUNE-1 and CNE-2 cells were plated at 500 cells per well in six-well plates after transfection, and cultured for 12 days. Colonies were fixed with methanol/acetic acid (3:1, v/v), stained with 0.5% crystal violet, and counted under the inverted microscope.

### In vitro migration and invasion assay

Transwell chambers (Corning) were coated without or with Matrigel (BD Biosciences) on the upper surface of membrane with 8 μm pore size, and used to test the cell migration or invasive ability. Briefly, 5 × 10^4^ or 1 × 10^5^ SUNE-1 and CNE-2 cells suspended in serum-free media were plated into the upper chamber for migration or invasion assay after transfection, and media supplemented with 10% FBS was placed into the lower chamber. After incubation for 8 h or 24 h, the cells that had migrated or invaded through the membrane to the lower surface were fixed, stained, and counted under the inverted microscope (100×).

### In vivo tumor growth model

Male BALB/c nude mice aged 4 to 6 weeks were purchased from the Hunan Slac Jingda Laboratory Animal Co., Ltd (Changsha, China). For tumor growth assay, SUNE-1 cells stably overexpression miR-451 or scramble miRNA were resuspended in PBS and 1 × 10^6^ cells (200 μl) were subcutaneously injected in the dorsal flank of nude mice. Tumor size was measured every 3 days and tumor volumes were calculated with the following formula: volume = (L × W^2^)/2, in which L meant the longest diameter and W meant the shortest diameter. Four weeks later, mice were sacrificed, and tumors were dissected and weighted. Animal handling and research protocols were approved by the Animal Care and Use Ethnic Committee.

### Luciferase reporter assay

The *MIF* wild-type (Wt) and mutant (Mt) 3′ UTR were created and cloned to the firefly luciferase-expressing vector psiCHECK™ (Promega). For the luciferase assay, SUNE-1 and CNE-2 cells were seeded in 6-well plates the day before transfection, and cotransfected with the *MIF* Wt or Mt 3′UTR reporter vector, the control vector pRL-TK (Promega), and miR-451 mimics or negative-control (NC) using Lipofectamine 2000 (Invitrogen). Luciferase activities were determined with the Dual-Luciferase Reporter System (Promega).

### Western blotting

Total cell proteins were separated using 9% SDS-PAGE gels, and transferred to PVDF (polyvinylidene fluoride) membranes (Millipore). Then, the membranes were incubated with mouse monoclonal anti-MIF antibody (1:1000; Abcam) followed by incubation with the HRP (horseradish peroxidase)-labeled goat anti-mouse IgG (1:2000; Boster). Bands were detected with chemiluminescence, and an anti-α-tubulin antibody (1:1000, Sigma) was used as the loading control.

### Statistical analysis

Data were presented as mean ± SD. The Student’s *t*-test, Chi-square test or Fisher’s exact test were used for comparisons between groups. The Kaplan-Meier method was used to estimate overall survival and disease-free survival, and multivariate Cox regression analysis with backward stepwise approach was used to test for independent prognostic factors. All statistical analysis was performed with SPSS 16.0 software, and *P* values of < 0.05 were defined as statistically significant.

## Competing interests

The authors declare that they have no competing interests.

## Authors’ contributions

NL, NJ, RG, WJ, QH, YX, and YL performed experiments; JM, NL, NJ, WJ, QH, YX, YL, LT, and YS designed research, analyzed data and edited the manuscript for intellectual content. All authors have made critical edits to the manuscript and have given final approval.
